# Efficacy of intra-articular hyaluronan (Synvisc^®^) for the treatment of osteoarthritis affecting the first metatarsophalangeal joint of the foot (hallux limitus): study protocol for a randomised placebo controlled trial

**DOI:** 10.1186/1757-1146-2-2

**Published:** 2009-01-16

**Authors:** Shannon E Munteanu, Hylton B Menz, Gerard V Zammit, Karl B Landorf, Christopher J Handley, Ayman ElZarka, Jason DeLuca

**Affiliations:** 1 Musculoskeletal Research Centre, Faculty of Health Sciences, La Trobe University, Bundoora 3086, Victoria, Australia; 2 Department of Podiatry, Faculty of Health Sciences, La Trobe University, Bundoora 3086, Victoria, Australia; 3 School of Human Biosciences, Faculty of Health Sciences, La Trobe University, Bundoora 3086, Victoria, Australia; 4 Southern Cross Medical Imaging, La Trobe University Private Hospital, Bundoora 3083, Victoria, Australia

## Abstract

**Background:**

Osteoarthritis of the first metatarsophalangeal joint (MPJ) of the foot, termed *hallux limitus*, is common and painful. Numerous non-surgical interventions have been proposed for this disorder, however there is limited evidence for their efficacy. Intra-articular injections of hyaluronan have shown beneficial effects in case-series and clinical trials for the treatment of osteoarthritis of the first metatarsophalangeal joint. However, no study has evaluated the efficacy of this form of treatment using a randomised placebo controlled trial. This article describes the design of a randomised placebo controlled trial to evaluate the efficacy of intra-articular hyaluronan (Synvisc^®^) to reduce pain and improve function in people with hallux limitus.

**Methods:**

One hundred and fifty community-dwelling men and women aged 18 years and over with hallux limitus (who satisfy inclusion and exclusion criteria) will be recruited.

Participants will be randomised, using a computer-generated random number sequence, to receive a single intra-articular injection of up to 1 ml hyaluronan (Synvisc^®^) or sterile saline (placebo) into the first MPJ. The injections will be performed by an interventional radiologist using fluoroscopy to ensure accurate deposition of the hyaluronan in the joint. Participants will be given the option of a second and final intra-articular injection (of Synvisc^® ^or sterile saline according to the treatment group they are in) either 1 or 3 months post-treatment if there is no improvement in pain and the participant has not experienced severe adverse effects after the first injection. The primary outcome measures will be the pain and function subscales of the Foot Health Status Questionnaire. The secondary outcome measures will be pain at the first MPJ (during walking and at rest), stiffness at the first MPJ, passive non-weightbearing dorsiflexion of the first MPJ, plantar flexion strength of the toe-flexors of the hallux, global satisfaction with the treatment, health-related quality of life (assessed using the Short-Form-36 version two questionnaire), magnitude of symptom change, use of pain-relieving medication and changes in dynamic plantar pressure distribution (maximum force and peak pressure) during walking. Data will be collected at baseline, then 1, 3 and 6 months post-treatment. Data will be analysed using the intention to treat principle.

**Discussion:**

This study is the first randomised placebo controlled trial to evaluate the efficacy of intra-articular hyaluronan (Synvisc^®^) for the treatment of osteoarthritis of the first MPJ (hallux limitus). The study has been pragmatically designed to ensure that the study findings can be implemented into clinical practice if this form of treatment is found to be an effective treatment strategy.

**Trial registration:**

Australian New Zealand Clinical Trials Registry: ACTRN12607000654459

## Background

Osteoarthritis (OA) is a degenerative joint disease that commonly presents within the first metatarsophalangeal joint (MPJ) of the foot. The terms *hallux limitus *and *hallux rigidus *have frequently been used interchangeably to describe differing severities of pain and limitation of motion associated with OA at the first MPJ [1]. *Hallux limitus *is a progressive osteoarthritic condition of the first MPJ that may advance to an end-stage presentation of *hallux rigidus *where the joint fuses and there is a complete restriction of motion [1]. First MPJ OA is the second most common disorder affecting the foot after hallux valgus [2]. The prevalence of the condition increases with age, and it has been reported that radiographic changes in the first MPJ affect are evident in approximately 46% of women and 32% of men at 60 years of age [3]. Osteoarthritis at the first MPJ is characterised by the symptoms of pain and stiffness at the joint [1]. Secondary painful symptoms relate to compensations during gait that may occur due to the reduced motion of the first MPJ [1]. The presence of pain associated with first MPJ OA impacts on normal walking and quality of life [4].

Treatment of hallux limitus involves conservative measures (such as physical therapy, foot orthoses, footwear modification, joint manipulation and injection with corticosteroid) [5], or surgical intervention (either joint-salvage or joint-destructive procedures) [6]. Pharmacological treatment is also often undertaken as an adjunct for pain relief in the management of hallux limitus [6]. However, although non-steroidal anti-inflammatory drugs (NSAIDs) and cyclooxygenase-2 inhibitors have been found to be effective in the management of various forms of OA, gastrointestinal complications remain a concern [7]. In light of these limitations with existing treatments, an alternative treatment termed 'viscosupplementation' – the intra-articular injection of hyaluronan into arthritic joints with the aim of restoring the viscoelasticity of the synovial fluid [8] – has been proposed and has attracted considerable attention in the medical literature as a treatment for OA [9]. In particular, both the American College of Rheumatology (ACR) and European League Against Rheumatism (EULAR) recommend hyaluronan in the management of OA of the knee [10,11]. Although the results of systematic reviews investigating the effectiveness of this type of treatment for knee OA are controversial, the most recent update of the Cochrane systematic review evaluating viscosupplementation for the treatment of knee OA concluded that viscosupplementation was both safe and effective for the treatment of OA and was superior or equivalent to any form of systemic intervention or intra-articular corticosteroids [9,12].

Despite there being a large number of studies investigating the effectiveness of hyaluronan for knee OA, few studies have investigated the effects of this form of treatment for OA at the first MPJ [13]. In a case-series retrospective study, 14 patients with radiographically confirmed OA at the first MPJ that received up to 3 intra-articular injections of 1 ml hyaluronan (Ostenil^® ^Mini) (sodium hyaluronate) reported a statistically significant reduction in pain (reported using a visual analogue scale) after 6 months [14]. The treatment was well tolerated, with 3/14 (21%) participants reporting mild adverse reactions at the injection site. In another study, Pons et al[13] compared a single intra-articular injection of 1 ml Ostenil^® ^Mini (sodium hyaluronate) with 1 ml Trigon depot^® ^(triamcinolone acetonide, a corticosteroid) for the treatment of painful, grade 1 hallux limitus (Karasick and Wapner [15] scale) in 37 participants (40 feet) [13]. Both treatment groups showed statistically significant reductions in pain at rest or on palpation for up to 12 weeks post-injection. However, hyaluronan treatment resulted in a statistically significant greater reduction in pain during walking and greater improvement in the American Orthopaedic Foot and Ankle Society (AOFAS) hallux MPJ score compared to treatment with triamcinolone acetonide. The treatment with hyaluronan was well tolerated, with 2/20 (10%) participants reporting mild adverse reactions at the injection site.

Although both of these studies suggest that intra-articular hyaluronan is safe and effective for the treatment of hallux limitus, neither used a placebo control group [13,14]. This limitation is significant as a placebo effect can account for 79% of the efficacy of intra-articular hyaluronan treatment [16]. Further, both studies are limited in that neither of the studies used blinding of both the participants and assessors in their protocols. It is therefore possible that the positive effects of hyaluronan may have been overestimated. Accordingly, the aims of this project are to conduct a double blind randomised controlled trial to determine the effectiveness of intra-articular hyaluronan (Synvisc^®^) on (i) foot pain and function; (ii) the range of motion of the first MPJ; (iii) the strength of the plantarflexor muscles of the first MPJ; (iv) the health related quality of life; and (v) the use of pain-relieving medications in people with hallux limitus. The study protocol is presented in this paper, consistent with the recommendations of Editorial Board of BioMed Central [17].

## Methods

### Design

**Figure 1:**
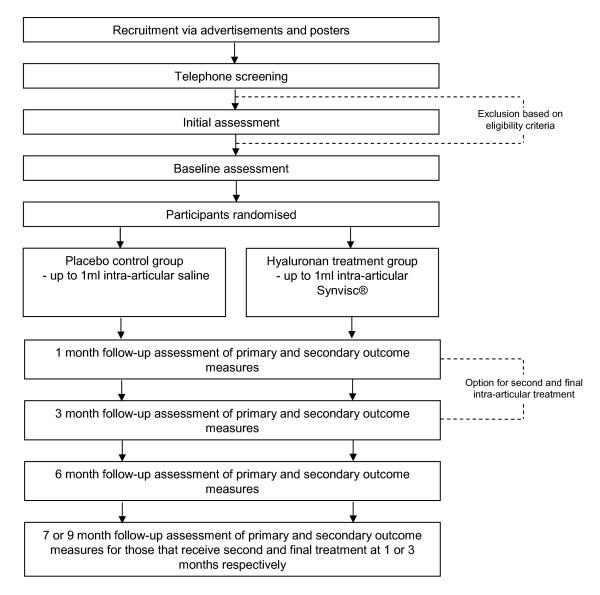
**Design of study**.

This study is a parallel group, participant and assessor blinded, randomised controlled trial with a 6 month follow-up (Figure 1). It has been developed using the principles described by Osteoarthritis Research Society International (OARSI) Clinical Trials Task Force guidelines [18]. Participants will be randomised to receive a single intra-articular injection of up to 1 ml hyaluronan (Synvisc^®^) or sterile saline (placebo) into the first MPJ. Allocation to either the Synvisc^® ^or placebo groups will be achieved using a computer-generated random number sequence. The allocation sequence will be generated and held by an external person not directly involved in the trial. Concealment of the allocation sequence will be ensured as each participant's allocation will be contained in a sealed opaque envelope. Envelopes will be made opaque by using a sheet of aluminium foil inside the envelope. In addition, a system using carbon paper will be employed so the details (name and date of recruitment) are transferred from the outside of the envelope to the paper inside the envelope containing the allocation prior to opening the seal. Assessors and participants will be blinded to group allocation. Participants will be given the option of a second and final intra-articular injection (of Synvisc^® ^or sterile saline according to the treatment group they are in) on days 30 or 90 if there is no improvement in pain and the participant has not experienced severe adverse effects after the first injection).

### Participants

The Human Studies Ethics Committee at La Trobe University (Human Ethics Committee Application No. 07-45) and the Radiation Advisory Committee of the Victorian Department of Human Services have given approval for the study. Written informed consent will be obtained from all participants prior to their participation. People with hallux limitus will be recruited from a number of sources:

(i) advertisements in relevant Melbourne (Australia) newspapers;

(ii) mail-out advertisements to health-care practitioners in Melbourne;

(iii) advertisements using relevant internet web-sites (including );

(iv) posters displayed in local retirement villages, community centres and universities located in Melbourne.

Respondents will initially be screened by telephone interview to ensure they are suitable for the study. Suitable individuals will then be invited to participate in the study and attend an initial assessment.

To be included in the study, participants must meet the following inclusion criteria:

(i) be aged at least 18 years;

(ii) report having symptoms of pain, during walking or rest, in the first MPJ for at least 3 months;

(iii) report having pain rated at least 20 mm on a 100 mm visual analogue pain scale (VAPS);

(iv) have pain upon palpation of the dorsal aspect of the first MPJ;

(v) radiographic evidence of OA (score 1 or 2 for either osteophytes or joint space narrowing using a previously published radiographic classification) [19] at the first MPJ.

(vi) able to walk household distances (>50 meters) without the aid of a walker, crutches or cane;

(vii) be willing to attend the La Trobe University Medical Centre (Melbourne, Australia) for treatment with either Synvisc^® ^or placebo (single intra-articular injection) and attend the Health Sciences Clinic at La Trobe University (Melbourne, Australia) for the initial assessment and the outcome measurements (at baseline and 1, 3 and 6 months post-treatment);

(viii) not receive other intra-articular injections into the first MPJ during the course of the study, apart from those dictated by the study;

(ix) be willing to discontinue taking all pain-relieving medications (analgesics and non-steroidal anti-inflammatory medications (NSAIDs), except paracetamol up to 4 g/day, taken by mouth or applied topically):

- for at least 14 days prior to the baseline assessment;

- during the study period (6 months after the final treatment with Synvisc^®^).

Participants who do take paracetamol need to discontinue its use at least 24 hours prior to the baseline assessment and follow-up assessments at 1, 3 and 6 months after the treatment;

(x) be willing to not receive any physical therapy on the involved MPJ or trial of shoe modifications or foot orthoses during the study period.

Exclusion criteria for participants in this study will be:

(i) Severe radiographic evidence of OA (score 3 for either osteophytes or joint space narrowing) at the first MPJ using a previously published radiographic classification [19];

(ii) previous surgery on the first MPJ;

(iii) intra-articular steroid, or any other intra-articular injection at the first MPJ in the previous 6 months;

(iv) treatment with systemic steroid (excluding inhalation or topical steroids), immunosuppressives or anticoagulants (except for acetylsalicylic acid at dosages of up to 325 mg/day);

(v) presence of joint infection(s) of the foot;

(vi) significant deformity of the first MPJ including hallux abducto valgus (grade of 3 or 4 scored using the Manchester Scale [20];

(vii) presence of peripheral vascular disease. Peripheral vascular disease will be considered to be present if any of the following are present [21];

▪ past history of, vascular surgery, Raynaud's phenomenon, vasculitis associated with connective tissue diseases, Buerger's disease, arterial emboli, deep vein thrombosis or lower limb ulcers;

▪ history of intermittent claudication or rest pain;

▪ presence of atrophy, ulcers or significant oedema;

▪ inability to palpate at least one pedal pulse;

▪ Ankle Brachial Pressure Index <0.9;

(viii) presence of one or more conditions that can confound pain and functional assessments of the first MPJ, such as metatarsalgia, plantar fasciitis, pre-dislocation syndrome, sprains of the foot, Achilles tendinopathy, degenerative joint disease of the foot (other than the first MPJ) or painful corns and callus;

(ix) planning to undergo any surgical procedure or receive any injections, apart from those dictated by the study, at the involved first MPJ during the study period;

(x) presence of systemic inflammatory condition or infection, such as inflammatory arthritis, diagnosed with rheumatoid arthritis, ankylosing spondylitis, psoriatic arthritis, reactive arthritis, septic arthritis, acute pseudogout, or any other connective tissue disease;

(xi) evidence of gout or other musculoskeletal disease other than OA within the feet. Gout will be screened for using clinical history and physical assessment (painful joint, abrupt onset, swelling), radiographic assessment (asymmetrical joint swelling, subcortical cysts without erosion and tophi) as well as serum uric acid levels (hyperuricaemia = serum uric acid > mean + 2 SD from normal population) [22];

(xii) active skin disease or infection in the area of the injection site;

(xiii) any medical condition that, in the opinion of the investigators, makes the participant unsuitable for inclusion (e.g., severe progressive chronic disease, malignancy, bleeding disorder, clinically important pain in a part of the musculoskeletal system other than the first MPJ, or fibromyalgia);

(xiv) pregnant or lactating women, or women who are of child bearing age or have not undergone menopause (Synvisc^® ^has not been tested in pregnant women or women who are nursing);

(xv) cognitive impairment (defined as a score of < 7 on the Short Portable Mental Status Questionnaire) [23];

(xvi) known hypersensitivity (allergy) to hyaluronan preparations, or to avian proteins, feathers or egg products;

(xvii) involvement in any clinical research study in the previous 3 months that could be considered to affect the results of this study.

### Intra-articular injections for the treatment groups

Participants will be randomised to receive a single intra-articular injection of up to 1 ml of hyaluronan (Synvisc^®^; Genzyme Biosurgery, Genzyme Corporation, NJ, USA) or sterile saline (placebo) into the first MPJ. Each 2 ml ampoule of Synvisc^® ^contains 16 mg of hylan G-F 20 (cross-linked hylan polymers; hylan A and B), 17 mg sodium chloride, 0.32 mg disodium hydrogen phosphate, 0.08 mg sodium dihydrogen phosphate monohydrate. The hyaluronan is extracted from chicken combs and the purified material has an average molecular weight of 6,000 kDa.

The injections will be performed by the same experienced interventional radiologist (AEZ) using fluoroscopic imaging to ensure accurate deposition of the hyaluronan within the joint. As the Synvisc^® ^is provided in ampoules that are labelled with the product name, it will not be possible to blind the injector, however this person is not involved in generation of the allocation order, recruitment, assessment or data analysis. The intra-articular injection will be performed using a 21 gauge (0.80 × 19 mm) Surflo^® ^(Terumo^® ^Corp., Tokyo, Japan) winged infusion set under aseptic procedures. Either a dorso-lateral or dorso-medial approach for injection will be used at the discretion of the injector (depending on which approach provides minimum interference from the osteophytes at the first MPJ joint margins). No anaesthetic will be used. If the participant has bilateral painful first MPJs, only one side (the most painful side) will be treated and used for data collection. The injector will record the volume of the agent that is injected.

Participants will be given the option of a second and final intra-articular injection (of Synvisc^® ^or sterile saline according to the treatment group they are in) on days 30 or 90 if there is no improvement in pain (assessed using the VAPS for pain during walking or at rest) and the participant has not experienced severe adverse effects after the first injection).

### Assessments

#### Initial assessments

An initial assessment will be performed to determine the eligibility of participants for this study. Demographic data will be collected including the age, gender, height and weight of participants. Data will also be obtained concerning the presentation of symptoms (foot affected, duration of symptoms). If the participant has bilateral painful first MPJs, the most painful side will be used for data collection and subsequent treatment. To establish eligibility for the study, participants will undergo a clinical assessment, have one set of dorso-plantar and lateral weight-bearing x-rays taken of their feet to grade the severity of first MPJ OA as well as undergo a blood test to assess serum uric acid levels (to exclude gout).

Weightbearing dorso-plantar and lateral radiographic views will be obtained from both feet with the participant standing in a relaxed bipedal stance position. All x-rays will be taken by the same medical imaging department using a Shimadzu UD150LRII 50 kw/30 kHz Generator and 0.6/1.2 P18DE-80S high speed x-ray tube from a ceiling suspended tube mount. AGFA MD40 CR digital phosphor plates in a 24 cm × 30 cm cassette will be used. For dorso-plantar projections, the x-ray tube will be angled 15° cephalad and centered at the base of the third metatarsal. For lateral projections, the tube will be angled 90° and centered at the base of the third metatarsal. The film focus distance will be set at 100 cm [19].

#### Baseline assessments and outcome measures

Participants who are eligible for the study will be invited to attend a baseline assessment. During the baseline assessment, participants will undergo primary and secondary outcome measurements prior to receiving their injection. The outcome measurements have been developed in accordance of the recommendations of the OARSI Clinical Trials Task Force guidelines [18].

#### Primary outcome measures

Outcome measurements (primary and secondary) will occur at four time-points at baseline, 1, 3 and 6 months post-treatment (after the intra-articular injection of Synvisc^® ^or placebo). The assessor performing the measurements will be blinded as to which treatment group participants have been allocated to. Participants who receive a second treatment at day 30 or 90 will be followed for a further 30 days or 90 days respectively and undergo outcome measurements at 7 or 9 months respectively.

The primary outcome measures will be the *Pain *and *Function *subscales of the Foot Health Status Questionnaire (FHSQ) [24]. The FHSQ includes 13 questions that assess four domains of foot health, *Foot pain, Foot function, Footwear *and *General foot health*. The FHSQ has been subjected to an extensive validation (content, criterion and construct validity) process. It has a high test-retest reliability (intraclass correlation coefficients ranging from 0.74 to 0.92) and a high degree of internal consistency (Cronbach's α ranging from 0.85 to 0.88) [24]. Rigorous reviews have rated it as one of the highest quality foot health status measures currently available [25-27].

#### Secondary outcome measures

The secondary outcome measures will be:

##### (i) Severity of pain

Severity of pain at the first MPJ during walking, and during rest, over the past week will be assessed using a 100 mm visual analogue pain scale. The left side of the scale (0 mm) will be labelled "no pain" and the right side of the scale (100 mm) will be labelled "worst pain possible" for each question [25,28].

##### (ii) Severity and duration of stiffness at the first metatarsophalangeal joint

The severity of stiffness at the first MPJ during walking over the past week will be assessed using a 100 mm visual analogue scale. The left side of the scale (0 mm) will be labelled "not stiff at all" and the right side of the scale (100 mm) will be labelled "most stiff possible". The average duration of stiffness at the first MPJ over the past week will be assessed using a four category scale response. The responses are: "none", "1–15 minutes", "16–30 minutes" and "greater than 30 minutes" [29].

##### (iii) Passive, non-weightbearing dorsiflexion range of motion of the first metatarsophalangeal joint

**Figure 2:**
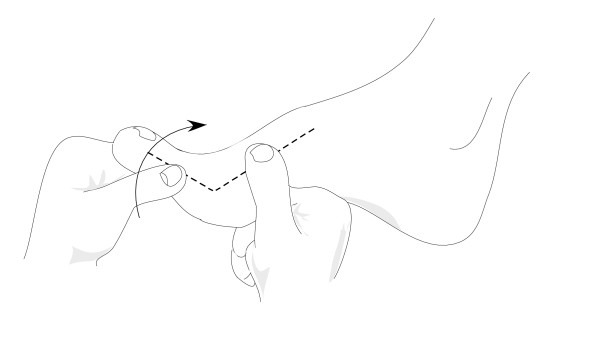
**Measurement of passive, non-weightbearing dorsiflexion range of motion of the first metatarsophalangeal joint**.

First MPJ dorsiflexion range of motion will be measured using a goniometer as the maximum angle at which the hallux cannot be passively moved into further extension in a non-weightbearing position (Figure 2) [30]. The test will be performed two times and the average will be used for analysis. This measurement technique shows high intra-reliability (ICC = 0.95, standard error of mean = 1.3°) [30].

##### (iv) Plantar flexion strength of the toe-flexors of the hallux

**Figure 3:**
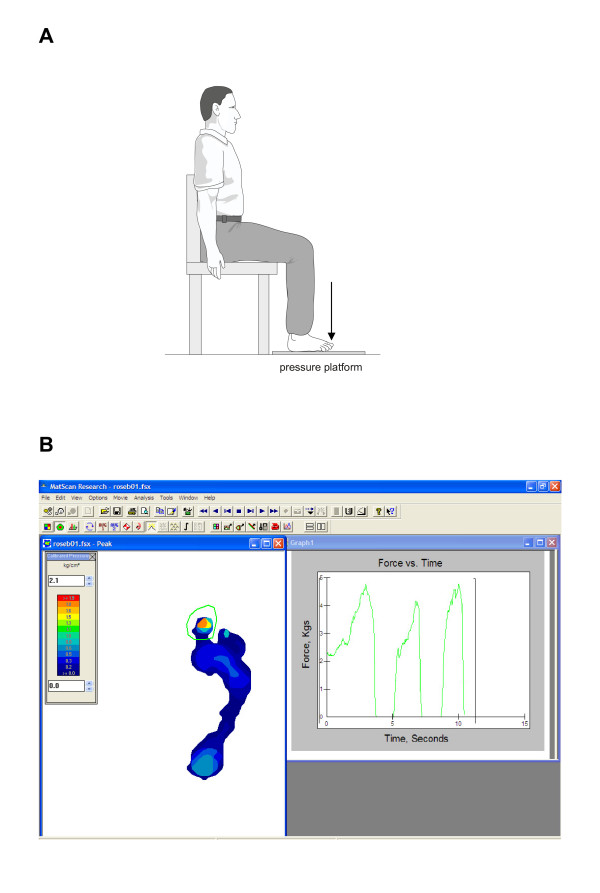
**Measurement of plantar flexion strength of the toe-flexors of the hallux. Participant positioning (A) and recording of forces produced under the hallux (B)**.

Plantar flexion strength of the toe-flexors of the hallux will be measured using the Mat Scan^® ^plantar pressure measurement device [31]. Participants will be seated with the hip, knee, and ankle at 90 degrees and their foot placed over the Mat Scan^® ^plantar pressure measurement device (Tekscan, Boston, MA, USA) (Figure 3a). This system consists of a 5-mm thick floor mat (432 × 368 mm) incorporating 2288 resistive sensors (1.4 sensors/cm2) sampling at a rate of 40 Hz. The mat will be calibrated for each participant using his or her own bodyweight before each testing session. Participants will be instructed to use their toe-flexor muscles to maximally push their hallux down on the MatScan^® ^device and forces under the hallux will be recorded (Figure 3b). The test will be performed three times for the hallux and the maximal force will be used for analysis. The test-retest reliability of this measurement technique has previously been shown to be high, with intraclass correlation coefficients (ICCs) = 0.88 (95% CI 0.81 – 0.93) [31].

##### (vi) Plantar pressure measurement

**Figure 4:**
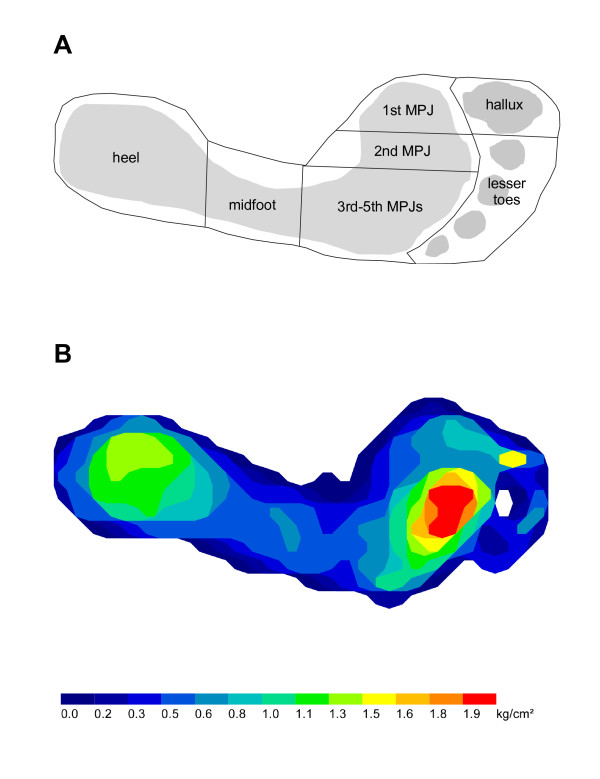
**The seven masked regions used for plantar pressure analysis (A) and typical plantar pressure recordings from a participant (B)**.

Plantar pressures will be recorded during level barefoot walking using the MatScan^® ^system (Tekscan^®^, Boston, MA, USA). The two-step gait initiation protocol will be used to obtain foot pressure data, as it requires fewer trials than the mid-gait protocol and has similar re-test reliability [32]. Three trials will be recorded, which has been found to be sufficient to ensure adequate reliability of pressure data [32,33]. Following data collection, the Research Foot^® ^software (version 5.24) will be used to construct individual "masks" to determine maximum force (kg) and peak pressure (kg/cm^2^) under seven regions of the foot: hallux, lesser toes, 1^st ^MPJ, 2^nd ^MPJ, 3^rd ^to 5^th ^MPJs, midfoot and heel (Figure 4a). For each region, the median of the three trials will be used for analysis. Typical plantar pressure recordings from a participant are shown in Figure 4b.

##### (vi) Global satisfaction with the treatment

Global satisfaction with the treatment will be assessed using a 5-point Likert scale, as well as a dichotomous (yes/no) scale. The five point-Likert scale will ask "How satisfied are you with the treatment you received for your big-toe joint pain?", and will have the following five responses: "Dissatisfied", "Only moderately satisfied", "Fairly satisfied", "Clearly satisfied" and "Very satisfied". The dichotomous scale of satisfaction will be answered as "Yes"' or "No" in response to the question: "Would you recommend the treatment that you received to someone else with big-toe joint pain".

##### (vii) Health related quality of life

The Short-Form-36 (version two) (SF-36) questionnaire will be used to assess health related quality of life. The SF-36 is a 36 question survey that measures eight health concepts most affected by disease and treatment. The eight health concepts can then be used to form two summary measures: *Physical health *and *Mental health*. The Short Form-36 (SF-36) has been extensively validated and is one of the most widely used instruments to measure health status. The SF-36 shows content, concurrent, criterion, construct, and predictive evidence of validity. The reliability of the eight concepts and two summary measures has been assessed using both internal consistency and test-retest methods. Reliability statistics have exceeded 0.80 [34-37].

##### (viii) Self-reported magnitude of symptom change

Self-reported magnitude of symptom change will be measured using a 15-point Likert scale. The scale will ask participants "how much have your symptoms in your big-toe joint have changed from the beginning of the study to now?". The fifteen responses will range from "A very great deal better" to "A very great deal worse".

##### (ix) Use of rescue medications to relieve pain at the first metatarsophalangeal joint

The number of participants who consumed rescue medication (e.g., paracetamol) and mean consumption of rescue medication to relieve pain at the first MPJ (mean grams of paracetamol/participant/month] will be assessed using a medications diary that participants will self-complete [38,39]. The diary will be returned to the assessor at monthly intervals for analysis.

##### (x) Frequency and severity of adverse events as safety variables

The frequency (number of participants affected and number of cases) and types of adverse events (including adverse drug reactions) in each treatment group during the trial will be recorded using a questionnaire that participants will complete during the follow-up appointments at 1, 3 and 6 months post-treatment [40]. To classify the 'type' of adverse event, a blinded assessor will classify the adverse event as being serious or non-serious [40]. Any serious adverse events, defined as adverse events leading to serious disability, hospital admission, or prolongation of hospitalisation, life-threatening events; or death) will be further classified using the International Classification of Diseases (ICD) codes [41]. Non-serious adverse events will include both local (pain, effusion and heat, with each classified as mild, moderate, severe) and systemic adverse events. An open-response type format will also be available for participant responses.

### Sample size

The sample size for the study has been pre-specified using an *a priori *power analysis using the primary outcome measure of the pain domain of the FHSQ [42]. One hundred and forty two participants (i.e. 71 per group) will provide power of 90% to detect a minimally important difference in the pain domain of the FHSQ (i.e. 14 points on the FHSQ questionnaire) with the significance level set at p < 0.05. A difference of 14 points was determined to be a clinically significant difference worth detecting [43] and a standard deviation of 25 was derived from a previous report [44]. This calculation included a 5% drop-out rate [13]. However, we will aim to recruit 150 participants (~75 participants per intervention group). Further, we have conservatively ignored the extra precision provided by covariate analysis when estimating the sample size.

### Statistical analysis

Statistical analysis will be undertaken using SPSS version 14.0 (SPSS Corp, Chicago, Ill, USA) and STATA 8 (Stata Corp, College Station, Tex., USA) statistical software. All analyses will be conducted on an intention-to-treat principle using all randomised participants [45-47]. Missing data will be replaced with the last score carried forward [48]. Standard tests for normal distribution will be used and transformation carried out if required.

Demographic characteristics (gender, age, weight, height, body mass index) will be determined for the baseline visit for each treatment group. Summary statistics will be calculated for duration of symptoms, side affected (left, right, bilateral), grade of OA at the first MPJ [19] as well as all primary and secondary outcome measurements for each treatment group.

Analyses will be conducted on 1, 3 and 6 month outcome measures. The continuously-scored outcome measures at 1, 3 and 6 months will be compared using analysis of covariance with baseline scores and intervention group entered as independent variables [49,50]. The exception to this will be the plantar pressure measurements which will be analysed at baseline, 1, 3 and 6 months using two-way repeated measures analysis of variance statistics. Post-hoc comparisons will be performed using Bonferroni-adjusted t-tests. Nominal and ordinal scaled data will be compared at 1, 3 and 6 months using Mann-Whitney U-tests and chi-square analyses (or Fisher's exact test where appropriate) respectively. Effect sizes will be determined using Cohen's *d *(continuous scaled data) or odds ratios (nominal scaled data and ordinal scaled data) as appropriate.

The outcome measurements obtained at 7 or 9 months for participants that receive a second and final intra-articular injection (of Synvisc^® ^or sterile saline according to the treatment group they are in) on days 30 or 90 respectively, will also be analysed as described above. These analyses will be classified as secondary outcomes.

## Discussion

This study is a randomised placebo controlled trial designed to investigate the efficacy of intra-articular hyaluronan (Synvisc^®^) to reduce pain and improve function in people with OA of the first MPJ (hallux limitus). Two studies have previously investigated the efficacy of intra-articular hyaluronan for the treatment of first MPJ OA [13,14]. However, neither of these studies used a placebo control group. To our knowledge, this is the first randomised controlled trial using intra-articular hyaluronan for OA of the first MPJ.

The use of a placebo control group is essential for studies evaluating the effects of intra-articular therapies as there is likely to be a large placebo response related to the injection procedure and this may inflate the results in uncontrolled evaluations [51]. Indeed, a recent meta-analysis of hyaluronan for knee OA concluded that a placebo effect accounted for 79% of the efficacy of intra-articular hyaluronan [16].

The study protocol and outcome measures have been developed in accordance of the recommendations of the OARSI Clinical Trials Task Force guidelines [18]. The outcome measures are pain and function subscales of the FHSQ, pain and stiffness at the first MPJ, range of motion (dorsiflexion) of the first MPJ, plantar flexion strength of muscles of the first MPJ, generic health related quality of life (SF-36), patient satisfaction with treatment, consumption of rescue medication as well as frequency and nature of adverse effects. These outcomes will be measured at baseline then at 1, 3 and 6 months after treatment. Previous research suggests that the effects of intra-articular hyaluronan persist for up to 12 months following treatment [9,38]. Thus, the use of follow-up assessments at 6 month post-treatment will allow us to determine if the effects, if any, of intra-articular hyaluronan persist in the longer term.

Participants will be given the option of a second and final intra-articular injection (of Synvisc^® ^or sterile saline according to the treatment group they are in) on days 30 or 90 if there is no improvement in their symptoms. Although this has the potential to complicate the interpretation of the results of the study, this protocol was included as it is likely to be more reflective of clinical practice [14], and this is in keeping with the pragmatic nature of this trial.

In summary, this project is the first randomised controlled trial to be conducted to evaluate the efficacy of intra-articular hyaluronan for reducing pain and improving function in people with hallux limitus. The study protocol, including interventions, have been pragmatically designed to ensure that the study findings are generaliseable to clinical practice. Recruitment for the study will commence in June 2008, and we expect final results to be available in mid-2010.

## Competing interests

HBM and KBL are Editor-in-Chief and Deputy Editor-in-Chief, respectively, of *Journal of Foot and Ankle Research*. It is journal policy that editors are removed from the peer review and editorial decision making processes for papers they have co-authored.

## Authors' contributions

SEM, HBM, KBL and CJH conceived the idea and obtained funding for the study. SEM, HBM, KBL, AEZ and JDL designed the trial protocol. SEM, HBM, KBL and GVZ drafted the manuscript. All authors have read and approved the final manuscript.

## References

[B1] Camasta C (1996). Hallux limitus and hallux rigidus. Clinical examination, radiographic findings, and natural history.. Clin Podiatr Med Surg.

[B2] Coughlin MJ, Shurnas PS (2003). Hallux rigidus: demographics, eitology and radiographic assessment. Foot Ankle Int.

[B3] van Saase JL, van Romunde LK, Cats A, Vandenbroucke JP, Valkenburg HA (1989). Epidemiology of osteoarthritis: Zoetermeer survey. Comparison of radiological osteoarthritis in a Dutch population with that in 10 other populations. Ann Rheum Dis.

[B4] Gorter K, Kuyvenhoven M, de Melker R (2000). Nontraumatic foot complaints in older people. A population-based survey of risk factors, mobility, and well-being. J Am Podiatr Med Assoc.

[B5] Weinfeld SB, Schon LC (1998). Hallux metatarsophalangeal arthritis. Clin Orthop Relat Res.

[B6] Vanore JV, Christensen JC, Kravitz SR, Schuberth JM, Thomas JL, Weil LS, Zlotoff HJ, Couture SD (2003). Diagnosis and treatment of First Metatarsophalangeal Joint Disorders. Section 2: Hallux rigidus. J Foot Ankle Surg.

[B7] Dugowson CE, Gnanashanmugam P (2006). Nonsteroidal anti-inflammatory drugs. Phys Med Rehabil Clin N Am.

[B8] Balazs EA, Denlinger JL (1993). Viscosupplementation: a new concept in the treatment of osteoarthritis. J Rheumatol Suppl.

[B9] Bellamy N, Campbell J, Robinson V, Gee T, Bourne R, Wells G (2006). Viscosupplementation for the treatment of osteoarthritis of the knee. Cochrane Database Syst Rev.

[B10] Pendleton A, Arden N, Dougados M, Doherty M, Bannwarth B, Bijlsma JWJ, Cluzeau F, Cooper C, Dieppe PA, Gunther K-P (2000). EULAR recommendations for the management of knee osteoarthritis: report of a task force of the Standing Committee for International Clinical Studies Including Therapeutic Trials (ESCISIT). Ann Rheum Dis.

[B11] None (2000). American College of Rheumatology Subcommittee on Osteoarthritis Guidelines: Recommendations for the medical management of osteoarthritis of the hip and knee: 2000 update. Arthritis Rheum.

[B12] Campbell J, Bellamy N, Gee T (2007). Differences between systematic reviews/meta-analyses of hyaluronic acid/hyaluronan/hylan in osteoarthritis of the knee. Osteoarthritis Cartilage.

[B13] Pons M, Alvarez F, Solana J, Viladot R, Varela L (2007). Sodium hyaluronate in the treatment of hallux rigidus. A single-blind, randomized study. Foot Ankle Int.

[B14] Maher A (2007). An audit of the use of sodium hyaluronate 1% (Ostenil^® ^Mini) therapy for the treatment of hallux rigidus. Br J Podiatry.

[B15] Karasick D, Wapner K (1991). Hallux rigidus deformity: radiologic assessment. Am J Roentgenol.

[B16] Lo GH, LaValley M, McAlindon T, Felson DT (2003). Intra-articular hyaluronic acid in treatment of knee osteoarthritis: A meta-analysis. JAMA.

[B17] Godlee F (2001). Publishing study protocols: Making them visible will improve registration, reporting and recruitment. BMC News and Views.

[B18] Altman R, Brandt K, Hochberg M, Moskowitz R, Bellamy N, Bloch DA, Buckwalter J, Dougados M, Ehrlich G, Lequesne M (1996). Design and conduct of clinical trials in patients with osteoarthritis: recommendations from a task force of the Osteoarthritis Research Society. Results from a workshop. Osteoarthritis Cartilage.

[B19] Menz HB, Munteanu SE, Landorf KB, Zammit GV, Cicuttini FM (2007). Radiographic classification of osteoarthritis in commonly affected joints of the foot. Osteoarthritis Cartilage.

[B20] Garrow AP, Papageorgiou A, Silman AJ, Thomas E, Jayson MIV, Macfarlane GJ (2001). The Grading of hallux valgus: The Manchester Scale. J Am Podiatr Med Assoc.

[B21] McLeod Roberts J, Merriman LM, Turner W (2002). Vascular Assessment. Assessment of the Lower Limb.

[B22] Zhang W, Doherty M, Bardin T, Pascual E, Barskova V, Conaghan P, Gerster J, Jacobs J, Leeb B, Liote F (2006). EULAR evidence based recommendations for gout. Part II: Management. Report of a task force of the EULAR Standing Committee For International Clinical Studies Including Therapeutics (ESCISIT). Ann Rheum Dis.

[B23] Pfeiffer E (1975). A short portable mental status questionnaire for the assessment of organic brain deficit in elderly patients. J Am Geriatr Soc.

[B24] Bennett P, Patterson C, Wearing S, Baglioni T (1998). Development and validation of a questionnaire designed to measure foot-health status. J Am Podiatr Med Assoc.

[B25] Landorf KB, Burns J, Yates B (2008). Health outcome assessment. Merriman's Assessment of the Lower Limb.

[B26] Martin RL, Irrgang JJ (2007). A Survey of Self-reported Outcome Instruments for the Foot and Ankle. J Orthop Sports Phys Ther.

[B27] Suk M, Hanson BP, Norvell DC, Helfet DL (2005). AO Handbook – Musculoskeletal Outcome Measures and Instruments.

[B28] McDowell I (2006). Measuring Health: A Guide to Rating Scales and Questionnaires.

[B29] Altman R, Asch E, Bloch D, Bole G, Borenstein D, Brandt K, Christy W, Cooke TD, Greenwald R, Hochberg M (1986). Development of criteria for the classification and reporting of osteoarthritis. Classification of osteoarthritis of the knee. Diagnostic and Therapeutic Criteria Committee of the American Rheumatism Association. Arthritis Rheum.

[B30] Hopson M, McPoil T, Cornwall M (1995). Motion of the first metatarsophalangeal joint. Reliability and validity of four measurement techniques. J Am Podiatr Med Assoc.

[B31] Menz HB, Zammit GV, Munteanu SE, Scott G (2006). Plantarflexion strength of the toes: age and gender differences and evaluation of a clinical screening test. Foot Ankle Int.

[B32] Bryant A, Singer K, Tinley P (1999). Comparison of the reliability of plantar pressure measurements using the two-step and midgait methods of data collection. Foot Ankle Int.

[B33] van der Leeden M, Dekker JH, Siemonsma PC, Lek-Westerhof SS, Steultjens MP (2004). Reproducibility of plantar pressure measurements in patients with chronic arthritis: a comparison of one-step, two-step, and three-step protocols and an estimate of the number of measurements required. Foot Ankle Int.

[B34] Ware J, Sherbourne C (1992). The MOS 36-item short-form health survey (SF-36). I. Conceptual framework and item selection. Med Care.

[B35] McHorney CA, Ware JE, Rogers W, Raczek AE, Lu JF (1992). The validity and relative precision of MOS short- and long-form health status scales and Dartmouth COOP charts. Results from the Medical Outcomes Study. Med Care.

[B36] McHorney CA, Ware JE, Raczek AE (1993). The MOS 36-Item Short-Form Health Survey (SF-36): II. Psychometric and clinical tests of validity in measuring physical and mental health constructs. Med Care.

[B37] McHorney CA, Ware JE, Lu JF, Sherbourne CD (1994). The MOS 36-item Short-Form Health Survey (SF-36): III. Tests of data quality, scaling assumptions, and reliability across diverse patient groups. Med Care.

[B38] Sun SF, Chou YJ, Hsu CW, Hwang CW, Hsu PT, Wang JL, Hsu YW, Chou MC (2006). Efficacy of intra-articular hyaluronic acid in patients with osteoarthritis of the ankle: a prospective study. Osteoarthritis Cartilage.

[B39] Lee PB, Kim YC, Lim YJ, Lee CJ, Sim WS, Ha CW, Bin SI, Lim KB, Choi SS, Lee SC (2006). Comparison between high and low molecular weight hyaluronates in knee osteoarthritis patients: Open-label, randomized, multicentre clinical trial. J Int Med Res.

[B40] Keech AC, Wonders SM, Cook DI, Gebski VJ (2004). Balancing the outcomes: reporting adverse events. Med J Aust.

[B41] Jüni P, Reichenbach S, Trelle S, Tschannen B, Wandel S, Jordi B, Züllig M, Guetg R, Häuselmann HJ, Schwarz H (2007). Efficacy and safety of intraarticular hylan or hyaluronic acids for osteoarthritis of the knee: a randomized controlled trial. Arthritis Rheum.

[B42] Friedman LM, Furberg CD, DeMets DL (1998). Fundamentals of Clinical Trials.

[B43] Landorf KB, Radford JA (2008). Minimal important difference: Values for the Foot Health Status Questionnaire, Foot Function Index and Visual Analogue Scale. The Foot.

[B44] Gilheany MF, Landorf KB, Robinson P (2008). Hallux valgus and hallux rigidus: a comparison of impact on health-related quality of life in patients presenting to foot surgeons in Australia. J Foot Ankle Res.

[B45] Gibaldi M, Sullivan S (1997). Intention-to-treat analysis in randomized trials: who gets counted?. J Clin Pharmacol.

[B46] Sheiner LB, Rubin DB (1995). Intention-to-treat analysis and the goals of clinical trials. Clin Pharmacol Ther.

[B47] Newell DJ (1992). Intention-to-treat analysis: Implications for quantitative and qualitative research. Int J Epidemiol.

[B48] Peat JK, Barton B (2005). Medical Statistics: A Guide to Data Analysis and Critical Appraisal.

[B49] Vickers AJ, Altman DG (2001). Statistics Notes: Analysing controlled trials with baseline and follow up measurements. BMJ.

[B50] Twisk J, Proper K (2004). Evaluation of the results of a randomized controlled trial: how to define changes between baseline and follow-up. J Clin Epidemiol.

[B51] Kirwan J (2001). Is there a place for intra-articular hyaluronate in osteoarthritis of the knee?. Knee.

